# Association between post-hospital clinic and telephone follow-up provider visits with 30-day readmission risk in an integrated health system

**DOI:** 10.1186/s12913-021-06848-9

**Published:** 2021-08-17

**Authors:** Huong Q. Nguyen, Aileen Baecker, Timothy Ho, Dan N. Huynh, Heather L. Watson, Jing Li, Ernest Shen

**Affiliations:** 1 Department of Research and Evaluation, Kaiser Permanente Southern California, Research and Evaluation, 100 S. Los Robles Avenue, 2nd Floor, Pasadena, CA 91101 USA; 2 Kaiser Permanente Southern California, Regional Clinical Operations, Pasadena, USA; 3 University of Kentucky, Lexington, USA

**Keywords:** Care transition, Readmission, Post-discharge provider follow-up

## Abstract

**Background:**

Follow-up visits with clinic providers after hospital discharge may not be feasible for some patients due to functional limitations, transportation challenges, need for physical distancing, or fear of exposure especially during the current COVID-19 pandemic.

**Methods:**

The aim of the study was to determine the effects of post-hospital clinic (POSH) and telephone (TPOSH) follow-up provider visits versus no visit on 30-day readmission. We used a retrospective cohort design based on data from 1/1/2017 to 12/31/2019 on adult patients (*n* = 213,513) discharged home from 15 Kaiser Permanente Southern California hospitals**.** Completion of POSH or TPOSH provider visits within 7 days of discharge was the exposure and all-cause 30-day inpatient and observation stay readmission was the primary outcome. We used matching weights to balance the groups and Fine-Gray subdistribution hazard model to assess for readmission risk.

**Results:**

Unweighted all-cause 30-day readmission rate was highest for patients who completed a TPOSH (17.3%) followed by no visit (14.2%), non-POSH (evaluation and management visits that were not focused on the hospitalization: 13.6%) and POSH (12.6%) visits. The matching weighted models showed that the effects of POSH and TPOSH visits varied across patient subgroups. For high risk (LACE 11+) medicine patients, both POSH (HR: 0.77, 95% CI: 0.71, 0.85, *P* < .001) and TPOSH (HR: 0.91, 95% CI: 0.83, 0.99, *P* = .03) were associated with 23 and 9% lower risk of 30-day readmission, respectively, compared to no visit. For medium to low risk medicine patients (LACE< 11) and all surgical patients regardless of LACE score or age, there were no significant associations for either visit type with risk of 30-day readmission.

**Conclusions:**

Post-hospital telephone follow-up provider visits had only modest effects on 30-day readmission in high-risk medicine patients compared to clinic visits. It remains to be determined if greater use and comfort with virtual visits by providers and patients as a result of the pandemic might improve the effectiveness of these encounters.

## Introduction

Transitional care management after hospital discharge typically includes a follow-up provider clinic visit within 7–14 days depending on patient complexity [1, 2]. We previously reported on the association between completion of a dedicated post-hospital (POSH) follow-up provider clinic visit with a 24% lower risk of 30-day inpatient readmissions in older patients within an integrated health system [3], a finding similar to other studies [4–6]. Nonetheless, since clinic visits were challenging for high risk patients who have functional limitations or no transportation, the health system began offering telephone POSH (TPOSH) appointments as an alternative to the clinic visits in 2015. Uptake of the TPOSH was limited and comprised only 7% of the post-discharge follow-up encounters prior to the COVID-19 pandemic.

In response to the dramatic shift to greater use of telehealth for routine clinic appointments out of necessity due to the COVID-19 pandemic [7], we aimed to advance the care transition evidence base by using data prior to the public health emergency to examine the effects of clinic (POSH) and telephone (TPOSH) visits completed within 7 days of discharge with a provider on 30-day inpatient and observation stay readmission compared to no visit and whether these effects varied by age, service line (medicine or surgical) and readmission risk as secondary analyses. In contrast to our earlier report [3], the analyses herein leverage a more contemporary cohort that reflects secular changes in transitional care management practices since 2014, is not restricted to older adults, and is not adversely affected by the disruptions in healthcare due to the COVID-19 pandemic.

## Methods

### Study design and sample

This retrospective cohort study included the first hospitalization for all adult patients who were discharged alive from 15 Kaiser Permanente Southern California (KPSC) hospitals between January 1, 2017 to December 31, 2019 to home or home health and remained enrolled in the health plan for at least 30 days post-discharge. This study was approved by the KPSC Institutional Review Board.

### Transitional care management

Continuous performance improvement efforts across the 15 hospitals focused on ensuring that follow-up provider visits were scheduled within 7 days of discharge for medicine high risk patients as determined by a LACE score [8] of 11 or higher during this study period; hospitals with greater resources were encouraged to also schedule appointments for medium risk (LACE score 7–10) patients. Most POSH and TPOSH visits were scheduled before hospital discharge with all sites consistently achieving > 85% appointments scheduled for high risk patients. TPOSH visits may be offered by hospital scheduling staff to patients who they anticipated would have challenges with completing a clinic POSH visit or were offered by call center staff if patients reported that they could not attend their POSH clinic appointment. POSH/TPOSH visits were flagged for the provider as being a post-hospital follow-up visit in the electronic medical record (EMR); reminders for routine care issues were suppressed during these visits. Providers were trained to focus on the post-discharge summary, medication review and issues that required follow-up. The POSH and TPOSH visits were generally 20 min in length. Other than the inability to conduct a physical assessment during a TPOSH, providers were expected to address standard transitional care needs of the patient. The TPOSH/POSH may take place before or after a standard 48–72-h post-discharge telephone call by an allied health staff. A non-POSH visit could have been scheduled before or after the hospitalization by the patient or a provider (primary care, specialist, and other clinicians) for any reason, including evaluation and management; the focus of these visits was not specifically on the hospitalization. Patients may have a follow-up visit with specialty care as a result of the hospitalization but due to challenges with access, these visits may not occur within the first 7 days of discharge.

### Covariates

Socio-demographic (age, gender, race/ethnicity, being partnered), behavioral (no-show history in the last 12 months), social risk (receipt of medical financial assistance from the health plan in the year prior to admission), and clinical characteristics [risk for readmission or early death score based on length of stay, age, comorbidities, and emergency department visits in the prior 6 months (LACE category [8]), severity of the index hospitalization (LAPS2) [9], discharge disposition (home vs. home health), service line (medicine vs. surgical), functional status (non-ambulatory vs. ambulatory), falls risk (Schmid [10] score of 3+) within 24 h of discharge, and frailty category [11]] were obtained or calculated from the electronic medical record (EMR) system.

### Outcome

The primary outcome was all-cause 30-day inpatient or observation stay readmission obtained from the EMR and claims.

### Statistical analysis

Although the rate of death in the overall sample was low (1.5%), the higher rate among the TPOSH group (3.6%) indicated a need to account for death as a competing risk, using the subdistribution hazard approach of Fine and Gray [12]. We treated visit completion as a time-dependent variable that could change in the first 7 days. While using time-varying covariates (i.e. visit completion) with the Fine-Gray subdistribution hazard model can preclude the estimation of their effect on either the subdistribution hazard or cumulative incidence function_,_ visit completion as we defined it is fixed for all subsequent risk sets, and so is not subject to this limitation [13].

We used matching weights [14] where the propensity score (PS) were calculated using generalized boosted models [15, 16] to address confounding instead of regression adjustment, inverse probability of treatment weights (IPTW) or matching approaches. We chose to use matching weights for several reasons: 1) it provides the average treatment effect on the treated (ATT) interpretation of switching patients from one exposure group to another (unlike IPTW which estimates average treatment effects on the entire sample); 2) it mitigates the bias that results from using IPTW when some groups have extreme weights such as we observed for the TPOSH group, in which case the substantial non-overlap on the PS [17] results in a violation of the common support assumption, by effectively assigning those with an extreme PS a weight close to 1; and 3) it avoids the nuances and complexity of matching on three or more groups to estimate the ATT, while also precluding the need to either exclude some patients from a matched analysis or introduce additional complexity by doing full matching [14, 18].

Covariates that were meaningfully associated with either visit completion or readmission [age, gender, race/ethnicity, being partnered, no-show history in the last 12 months, receipt of medical financial assistance in the year prior to admission (a marker of social risk), risk for readmission or early death (LACE category [8]), severity of hospitalization (LAPS2) [9], discharge disposition (home vs. home health), service line (medicine vs. surgical), functional status (non-ambulatory vs. ambulatory) and fall risk (Schmid [10] score of 3+) within 24 h of discharge, frailty category [11], and hospital site] were included in the multinomial regression models to estimate propensity scores to construct the matching weights. Such associations were deemed meaningful using a combination of prior literature and examining statistical measures of association (namely the maximum standardized pairwise differences). Secondary subgroup analyses were stratified by age (< 65 or 65+), service line (medicine vs. surgical) and LACE score (< 11: medium to low risk vs. 11+: high risk), to assess for heterogeneity of treatments effects. Analyses were performed with SAS Enterprise Guide 7.1 (Cary, North Carolina). A *P* < .05 was considered significant.

## Results

### Patient flow

**Fig. 1:**
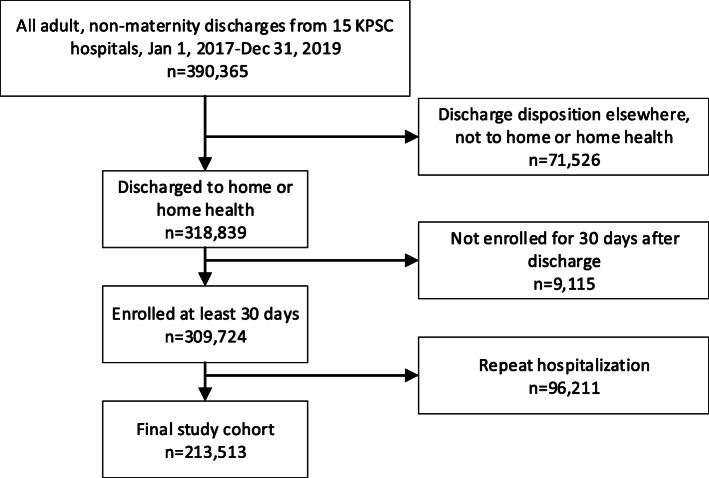
Sample Flow

The initial cohort of 390,365 adult, non-maternity patients discharged alive from 15 hospitals was pared down to a total of 213,513 unique patients who had their first index hospitalization during the study period after excluding 71,526 patients due to discharge disposition elsewhere not to home or home health, 9115 patients not enrolled for at least 30 days post discharge, and 96,211 patients with repeat hospitalizations during the study period (Fig. 1).

### Sample characteristics

Approximately 30% of patients completed a POSH (26%) or TPOSH (4%) visit within 7-days of hospital discharge; 33% had a non-POSH visit and 37% did not have any clinic visit (Tables 1 and 2). Approximately 57% of the high-risk medicine LACE patients for whom the hospitals prioritized for scheduling follow-up appointments completed a POSH or TPOSH visit within 7 days whereas 25% of the medium to low-risk medicine LACE patients completed such visits. Patients who completed a TPOSH tended to be older, had higher co-morbidities, frailty and readmission risk compared to patients completing other visit types or no visit. Surgical patients were more likely to complete nonPOSH visits or no visit compared to medicine patients. The maximum standardized differences in matching weights across all baseline variables were all < 0.10 indicating acceptable balance across all six possible pairwise comparisons (POSH vs. no visit, TPOSH vs. no visit, nonPOSH vs. no visit, POSH vs. TPOSH, POSH vs. nonPOSH, TPOSH vs. nonPOSH) [19].

**Table 1:** Baseline sample characteristics by completion of post-hospital follow-up provider visits within 7-days of discharge

	POSH	TPOSH	Non-POSH	No Visit	Total	Max Std Diff	
	***n*** = 56,383 (26.4%)	***n*** = 9009 (4.2%)	***n*** = 69,997 (32.8%)	***n*** = 78,124 (36.6%)	***n*** = 213,513	Not Wgtd	Wgtd
**Socio-demographics**							
Age	66.5 (16.4)	69.5 (16.2)	58.9 (17.8)	57.8 (18.6)	60.9 (18.1)	0.68	0.05
Female	27,203 (48.2%)	4857 (53.9%)	36,903 (52.7%)	45,197 (57.9%)	114,160 (53.5%)	0.10	0.01
Race/Ethnicity							
Asian	5042 (8.9%)	721 (8%)	6432 (9.2%)	6459 (8.3%)	18,654 (8.7%)	0.01	0.00
Black	6440 (11.4%)	1446 (16.1%)	7434 (10.6%)	9146 (11.7%)	24,466 (11.5%)	0.05	0.01
Hispanic	18,788 (33.3%)	2517 (27.9%)	24,815 (35.5%)	28,760 (36.8%)	74,880 (35.1%)	0.09	0.01
Other	1041 (1.8%)	155 (1.7%)	1385 (2%)	1554 (2%)	4135 (1.9%)	0.00	0.00
White	25,072 (44.5%)	4170 (46.3%)	29,931 (42.8%)	32,205 (41.2%)	91,378 (42.8%)	0.05	0.01
Marital status: Partnered	32,842 (58.2%)	4622 (51.3%)	42,151 (60.2%)	44,166 (56.5%)	123,781 (58%)	0.09	0.01
Education: < College	41,216 (73.1%)	6504 (72.2%)	50,048 (71.5%)	56,640 (72.5%)	154,370 (72.3%)	0.09	0.05
Household income: >$20,000	48,602 (86.2%)	7667 (85.1%)	60,477 (86.4%)	67,187 (86.0%)	184,048 (86.2%)	0.15	0.06
Spoken language: English	48,993 (86.9%)	8108 (90%)	62,222 (88.9%)	69,453 (88.9%)	188,776 (88.4%)	0.03	0.02
Insurance type^a^							
Commercial/private pay	21,503 (38.1%)	2772 (30.8%)	37,651 (53.8%)	43,070 (55.1%)	104,996 (49.2%)	0.24	0.02
Dual	3215 (5.7%)	591 (6.6%)	2450 (3.5%)	2907 (3.7%)	9163 (4.3%)	0.03	0.01
Medicaid	2493 (4.4%)	392 (4.4%)	3838 (5.5%)	4754 (6.1%)	11,477 (5.4%)	0.02	0.01
Medicare	29,160 (51.7%)	5250 (58.3%)	26,035 (37.2%)	27,330 (35%)	87,775 (41.1%)	0.23	0.02
Received medical financial assistance in prior year	4590 (8.1%)	941 (10.4%)	5163 (7.4%)	5035 (6.4%)	15,729 (7.4%)	0.04	0.00
**Health and Behavior Before Hospitalization**							
Clinic no show rate (at least once)	34,084 (60.5%)	6185 (68.7%)	43,594 (62.3%)	47,297 (60.5%)	131,160 (61.4%)	0.08	0.01
Charlson comorbidity index^b^	4.7 (3.08)	5.5 (3.14)	3.8 (2.99)	3.4 (2.94)	4.0 (3.07)	0.68	0.03
Myocardial infarction	9635 (17.1%)	1727 (19.2%)	7030 (10%)	6703 (8.6%)	25,095 (11.8%)	–	–
Congestive heart failure	13,396 (23.8%)	2684 (29.8%)	9295 (13.3%)	8613 (11%)	33,988 (15.9%)	–	–
Peripheral vascular disease	26,973 (47.8%)	5236 (58.1%)	22,050 (31.5%)	22,215 (28.4%)	76,474 (35.8%)	–	–
Cerebrovascular disease	12,175 (21.6%)	2358 (26.2%)	9105 (13%)	9833 (12.6%)	33,471 (15.7%)	–	–
Dementia	3536 (6.3%)	1134 (12.6%)	1940 (2.8%)	3357 (4.3%)	9967 (4.7%)	–	–
Rheumatic disease	3937 (7%)	710 (7.9%)	3692 (5.3%)	3711 (4.8%)	12,050 (5.6%)	–	–
Peptic ulcer disease	3779 (6.7%)	687 (7.6%)	3094 (4.4%)	3296 (4.2%)	10,856 (5.1%)	–	–
Mild liver disease	8727 (15.5%)	1499 (16.6%)	9095 (13%)	9301 (11.9%)	28,622 (13.4%)	–	–
Moderate or severe liver disease	1355 (2.4%)	236 (2.6%)	890 (1.3%)	837 (1.1%)	3318 (1.6%)	–	–
Diabetes without chronic complication	7821 (13.9%)	1159 (12.9%)	8766 (12.5%)	9464 (12.1%)	27,210 (12.7%)	–	–
Diabetes with chronic complication	18,284 (32.4%)	3201 (35.5%)	14,142 (20.2%)	12,878 (16.5%)	48,505 (22.7%)	–	–
Hemiplegia or paraplegia	2909 (5.2%)	740 (8.2%)	2211 (3.2%)	2721 (3.5%)	8581 (4%)	–	–
Renal disease	20,089 (35.6%)	3776 (41.9%)	14,696 (21%)	14,609 (18.7%)	53,170 (24.9%)	–	–
Any malignancy (lymphomas, leukemias etc)	8825 (15.7%)	1788 (19.8%)	11,963 (17.1%)	9563 (12.2%)	32,139 (15.1%)	–	–
Metastatic solid tumor	3343 (5.9%)	830 (9.2%)	4578 (6.5%)	3006 (3.8%)	11,757 (5.5%)	–	–
AIDS/HIV	236 (0.4%)	51 (0.6%)	281 (0.4%)	244 (0.3%)	812 (0.4%)	–	–
Frailty index	8.8 (6.65)	11.1 (8.35)	6.1 (6.05)	5.7 (6.44)	6.9 (6.65)		
Low risk < 5	18,052 (32%)	2310 (25.6%)	37,893 (54.1%)	46,855 (60%)	105,110 (49.2%)	0.34	0.01
Intermediate risk 5–14	29,643 (52.6%)	4366 (48.5%)	26,225 (37.5%)	24,610 (31.5%)	84,844 (39.7%)	0.21	0.01
High Risk 15+	8688 (15.4%)	2333 (25.9%)	5879 (8.4%)	6659 (8.5%)	23,559 (11%)	0.17	0.01

Values are presented as either mean (SD) or n (column %), unless otherwise indicated

*POSH* Post-hospital follow-up clinic visit, *TPOSH* Post-hospital follow-up telephone visit, *nonPOSH* visit not related to post-hospital follow-up

*Wgtd* Weighted maximum standardized differences across groups

^a^ Missingness < 0.1

^b^Weighting only done on index, not individual conditions

**Table 2:** Characteristics of the index hospitalization, 30-day readmission and mortality

	POSH	TPOSH	Non-POSH	No Visit	Total	Max Std Diff	
	***n*** = 56,383 (26.4%)	***n*** = 9009 (4.2%)	***n*** = 69,997 (32.8%)	***n*** = 78,124 (36.6%)	***n*** = 213,513	Not Wgtd	Wgtd
All cause 30-day inpatient or observation stay readmission	7093 (12.6%)	1562 (17.3%)	9540 (13.6%)	11,093 (14.2%)	29,288 (13.7%)	–	–
30-day mortality	605 (1.1%)	324 (3.6%)	683 (1.0%)	1503 (1.9%)	3115 (1.5%)	–	–
**Index Hospitalization**							
Source of admission^a^							
Home/clinic	46,209 (82%)	7297 (81%)	60,593 (86.6%)	68,386 (87.5%)	182,485 (85.5%)	0.09	0.02
Hospital/SNF	9912 (17.6%)	1628 (18.1%)	8748 (12.5%)	9157 (11.7%)	29,445 (13.8%)	0.06	0.03
Other	204 (0.4%)	45 (0.5%)	608 (0.9%)	493 (0.6%)	1350 (0.6%)	0.01	0.00
Service line							
Surgical	9775 (17.3%)	1773 (19.7%)	40,411 (57.7%)	48,886 (62.6%)	100,845 (47.2%)	0.45	0.03
Medicine	46,608 (82.7%)	7236 (80.3%)	29,586 (42.3%)	29,238 (37.4%)	112,668 (52.8%)	–	–
Major diagnostic categories *(row %)*							
Diseases/Disorders of the Circulatory System	11,626 (37.6%)	1623 (5.2%)	10,512 (34%)	7173 (23.2%)	30,934 (14.5%)	0.11	0.05
Diseases/Disorders of the Musculoskeletal System	2421 (8.5%)	696 (2.5%)	9188 (32.4%)	16,049 (56.6%)	28,354 (13.3%)	0.16	0.05
Infectious & Parasitic Diseases, Systemic or Unspecified Sites	11,584 (42.4%)	1845 (6.7%)	6574 (24%)	7343 (26.9%)	27,346 (12.8%)	0.11	0.04
Diseases/Disorders of the Digestive System	6672 (27.5%)	977 (4%)	7443 (30.7%)	9187 (37.8%)	24,279 (11.4%)	0.01	0.02
Diseases/Disorders of the Nervous System	4946 (33.2%)	595 (4%)	4431 (29.7%)	4940 (33.1%)	14,912 (7%)	0.02	0.03
Endocrine, Nutritional & Metabolic Diseases & Disorders	1922 (15.4%)	362 (2.9%)	3528 (28.3%)	6635 (53.3%)	12,447 (5.8%)	0.05	0.01
Diseases/Disorders of the Respiratory System	4985 (40.7%)	822 (6.7%)	3111 (25.4%)	3317 (27.1%)	12,235 (5.7%)	0.05	0.02
Diseases/Disorders of the Hepatobiliary System	3212 (27.4%)	420 (3.6%)	3373 (28.7%)	4739 (40.4%)	11,744 (5.5%)	0.01	0.01
Laboratory Acute Physiology Score (LAPS2)	70.1 (30.42)	73.4 (31.94)	57.8 (27.98)	56.2 (28.43)	61.1 (29.66)	0.58	0.03
LACE readmission risk score	10.4 (2.80)	11.2 (2.93)	8.0 (3.59)	7.0 (3.66)	8.4 (3.70)		
LACE < 11	26,301 (46.6%)	2810 (31.2%)	52,055 (74.4%)	64,181 (82.2%)	145,347 (68.1%)	0.51	0.02
LACE 11 to 15	28,897 (51.3%)	5787 (64.2%)	16,928 (24.2%)	12,997 (16.6%)	64,609 (30.3%)	0.48	0.04
LACE 16 to 19	1185 (2.1%)	412 (4.6%)	1014 (1.4%)	946 (1.2%)	3557 (1.7%)	0.03	0.01
Length of stay							
0–3 days	29,460 (52.2%)	4044 (44.9%)	44,692 (63.8%)	57,202 (73.2%)	135,398 (63.4%)	0.28	0.05
4–6 days	17,659 (31.3%)	2998 (33.3%)	16,312 (23.3%)	14,789 (18.9%)	51,758 (24.2%)	0.14	0.04
7–13 days	7491 (13.3%)	1427 (15.8%)	7221 (10.3%)	4913 (6.3%)	21,052 (9.9%)	0.10	0.02
14+ days	1773 (3.1%)	540 (6%)	1772 (2.5%)	1220 (1.6%)	5305 (2.5%)	0.04	0.02
Functional status within 24 h of discharge							
Non-ambulatory	6177 (11%)	2231 (24.8%)	7213 (10.3%)	11,525 (14.8%)	27,146 (12.7%)	0.14	0.01
Ambulatory with assistance	27,514 (48.8%)	4057 (45%)	27,458 (39.2%)	29,276 (37.5%)	88,305 (41.4%)	0.11	0.00
Ambulatory	21,965 (39%)	2569 (28.5%)	28,967 (41.4%)	30,000 (38.4%)	83,501 (39.1%)	0.13	0.01
Missing	727 (1.3%)	152 (1.7%)	6359 (9.1%)	7323 (9.4%)	14,561 (6.8%)	0.08	0.00
Schmid fall risk score 3+ within 24 h of discharge	9354 (16.6%)	2107 (23.4%)	11,882 (17%)	14,608 (18.7%)	37,951 (17.8%)	0.07	0.01
Discharge disposition							
Home	36,726 (65.1%)	4755 (52.8%)	49,184 (70.3%)	55,429 (71%)	146,094 (68.4%)	0.18	0.01
Home health	19,657 (34.9%)	4254 (47.2%)	20,813 (29.7%)	22,695 (29%)	67,419 (31.6%)	–	–

Values are presented as either mean (SD) or n (column %), unless otherwise indicated

*POSH* Post-hospital follow-up clinic visit, *TPOSH* Post-hospital follow-up telephone visit, *nonPOSH* visit not related to post-hospital follow-up

*Wgtd* Weighted maximum standardized differences across groups

^a^ Missingness < 0.1

A total of 29,288 (13.7%) and 3115 (1.5%) of patients were readmitted or died within 30 days of being discharged alive, respectively. Among those patients who died within 30 days, 58% had a readmission prior to death, while the remainder had a median survival time of 13 days (IQR: 6, 21). The unweighted all-cause 30-day readmission rate was highest for patients who completed a TPOSH (17.3%) followed by no visit (14.2%), non-POSH (13.6%) and POSH (12.6%) (Table 2).

### Overall primary analyses

For all patients, only POSH (HR: 0.83, 95% CI: 0.77, 0.90, *P* < .001) provider visits completed within 7 days of hospital discharge were associated with a 17% lower risk of 30-day inpatient or observation readmission compared to no visit in the weighted models (Table 3). TPOSH provider visits were not associated with reductions in readmission risk for all patients (HR: 0.97, 95% CI: 0.90, 1.05, *P* = .48). POSH provider visits were associated with 15% lower risk of 30-day readmission compared to TPOSH provider visits (HR: 0.85, 95% CI: 0.79, 0.93, *P* < .01).

**Table 3:** Weighted models examining the effect of post-hospital follow-up provider visits completed within 7-days of discharge on all cause 30-day inpatient and observation stay readmission stratified by age, service line, and readmission risk

	All patients		Age: 65+		Age < 65	
	***n*** = 213,513		***n*** = 102,011		***n*** = 111,502	
	Crude HR (95%CI)	Weighted HR (95%CI)	Crude HR (95%CI)	Weighted HR (95%CI)	Crude HR (95%CI)	Weighted HR (95%CI)
**All Patients**						
POSH vs. no visit	1.34 (1.3, 1.38)	0.83 (0.77, 0.9)	1.17 (1.12, 1.22)	0.83 (0.76, 0.92)	1.38 (1.31, 1.46)	0.82 (0.71, 0.95)
TPOSH vs. no visit	1.85 (1.74, 1.95)	0.97 (0.9, 1.05)	1.51 (1.41, 1.62)	0.94 (0.86, 1.03)	2.10 (1.9, 2.32)	1.04 (0.92, 1.19)
nonPOSH vs. no visit	1.37 (1.33, 1.41)	1.05 (0.98, 1.13)	1.15 (1.11, 1.2)	1.08 (1.00, 1.18)	1.59 (1.53, 1.66)	0.98 (0.86, 1.12)
POSH vs. TPOSH	0.73 (0.68, 0.77)	0.85 (0.79, 0.93)	0.77 (0.72, 0.83)	0.89 (0.8, 0.98)	0.66 (0.59, 0.73)	0.79 (0.68, 0.91)
**Medicine,*n*** **= 112,668**						
POSH vs. no visit	0.91 (0.88, 0.95)	0.79 (0.73, 0.86)	0.85 (0.81, 0.89)	0.81 (0.73, 0.89)	0.95 (0.89, 1.01)	0.75 (0.65, 0.88)
TPOSH vs. no visit	1.31 (1.23, 1.39)	0.95 (0.87, 1.02)	1.13 (1.05, 1.22)	0.93 (0.85, 1.02)	1.54 (1.38, 1.73)	0.98 (0.85, 1.13)
nonPOSH vs. no visit	1.14 (1.09, 1.18)	1.04 (0.96, 1.12)	1.11 (1.05, 1.17)	1.07 (0.98, 1.17)	1.19 (1.12, 1.26)	0.95 (0.82, 1.09)
POSH vs. TPOSH	0.7 (0.65, 0.74)	0.84 (0.77, 0.91)	0.75 (0.69, 0.80)	0.87 (0.78, 0.96)	0.61 (0.55, 0.69)	0.77 (0.65, 0.9)
**LACE 11+ (High risk)**						
POSH vs. no visit	0.73 (0.69, 0.76)	0.77 (0.71, 0.85)	0.75 (0.71, 0.80)	0.79 (0.71, 0.88)	0.67 (0.62, 0.74)	0.72 (0.61, 0.86)
TPOSH vs. no visit	0.92 (0.86, 0.99)	0.91 (0.83, 0.99)	0.94 (0.87, 1.03)	0.92 (0.83, 1.02)	0.87 (0.76, 1.00)	0.87 (0.74, 1.03)
nonPOSH vs. no visit	0.99 (0.94, 1.04)	1.01 (0.93, 1.09)	1.04 (0.97, 1.11)	1.05 (0.96, 1.16)	0.89 (0.81, 0.97)	0.90 (0.77, 1.05)
POSH vs. TPOSH	0.79 (0.74, 0.85)	0.85 (0.77, 0.94)	0.80 (0.74, 0.87)	0.86 (0.77, 0.97)	0.78 (0.68, 0.89)	0.82 (0.69, 0.99)
**LACE < 11 (Medium/low risk)**						
POSH vs. no visit	0.87 (0.82, 0.93)	0.84 (0.67, 1.05)	0.83 (0.76, 0.91)	0.86 (0.65, 1.15)	0.86 (0.78, 0.95)	0.79 (0.55, 1.13)
TPOSH vs. no visit	1.23 (1.06, 1.43)	1.14 (0.94, 1.39)	1.01 (0.83, 1.24)	1.02 (0.78, 1.33)	1.44 (1.15, 1.80)	1.32 (0.98, 1.78)
nonPOSH vs. no visit	1.21 (1.14, 1.29)	1.16 (0.96, 1.42)	1.16 (1.06, 1.27)	1.19 (0.92, 1.54)	1.27 (1.17, 1.39)	1.15 (0.85, 1.56)
POSH vs. TPOSH	0.71 (0.61, 0.82)	0.73 (0.58, 0.92)	0.82 (0.67, 1.01)	0.85 (0.63, 1.14)	0.59 (0.47, 0.75)	0.60 (0.42, 0.85)
**All Surgical*n*** **= 100,845**						
POSH vs. no visit	1.35 (1.24, 1.46)	1.01 (0.8, 1.28)	1.21 (1.08, 1.35)	0.93 (0.69, 1.25)	1.34 (1.19, 1.51)	1.11 (0.76, 1.63)
TPOSH vs. no visit	1.68 (1.43, 1.96)	1.17 (0.94, 1.45)	1.48 (1.20, 1.83)	1.05 (0.79, 1.39)	1.68 (1.31, 2.15)	1.34 (0.94, 1.89)
nonPOSH vs. no visit	1.65 (1.57, 1.73)	1.07 (0.86, 1.34)	1.17 (1.08, 1.27)	1.01 (0.76, 1.36)	1.93 (1.82, 2.05)	1.18 (0.83, 1.67)
POSH vs. TPOSH	0.81 (0.68, 0.96)	0.87 (0.68, 1.10)	0.82 (0.65, 1.02)	0.89 (0.66, 1.20)	0.80 (0.61, 1.05)	0.83 (0.57, 1.21)
**LACE 11+ (High risk)**						
POSH vs. no visit	0.73 (0.64, 0.84)	0.77 (0.56, 1.07)	0.75 (0.63, 0.89)	0.80 (0.54, 1.16)	0.72 (0.58, 0.89)	0.72 (0.39, 1.32)
TPOSH vs. no visit	1.11 (0.88, 1.38)	1.1 (0.82, 1.47)	1.12 (0.84, 1.48)	1.05 (0.74, 1.49)	1.11 (0.76, 1.62)	1.20 (0.71, 2.03)
nonPOSH vs. no visit	0.95 (0.84, 1.06)	0.95 (0.7, 1.29)	0.93 (0.8, 1.09)	0.97 (0.67, 1.41)	0.95 (0.80, 1.13)	0.90 (0.52, 1.55)
POSH vs. TPOSH	0.66 (0.53, 0.84)	0.7 (0.51, 0.97)	0.67 (0.50, 0.90)	0.76 (0.51, 1.12)	0.64 (0.44, 0.95)	0.60 (0.33, 1.07)
**LACE < 11 (Medium/low risk)**						
POSH vs. no visit	1.21 (1.08, 1.36)	1.36 (0.96, 1.92)	1.21 (1.02, 1.43)	1.24 (0.76, 2.03)	1.14 (0.97, 1.34)	1.43 (0.87, 2.37)
TPOSH vs. no visit	1.3 (1.03, 1.65)	1.28 (0.91, 1.80)	1.22 (0.88, 1.70)	1.17 (0.72, 1.88)	1.27 (0.9, 1.80)	1.37 (0.84, 2.22)
nonPOSH vs. no visit	1.75 (1.66, 1.85)	1.30 (0.92, 1.82)	1.20 (1.09, 1.33)	1.17 (0.72, 1.92)	2.02 (1.9, 2.15)	1.42 (0.89, 2.27)
POSH vs. TPOSH	0.93 (0.72, 1.21)	1.06 (0.75, 1.49)	0.99 (0.69, 1.41)	1.07 (0.67, 1.70)	0.90 (0.62, 1.31)	1.05 (0.64, 1.73)

Values are presented as hazard ratio (HR) and 95% confidence intervals

*POSH* Post-hospital follow-up clinic visit, *TPOSH* Telephone post-hospital follow-up visit, *nonPOSH* Visit not related to post-hospital follow-up, *LACE* Length of stay; Acuity of admission; Co-morbidities; Emergency visits in previous 6 months

Matching weights were used, based on multinomial propensity score models that included age, gender, race/ethnicity, being partnered, no-show history in the last 12 months, receipt of medical financial assistance in the year prior to admission (a marker of social risk), frailty category, risk for readmission or early death (LACE category), severity of hospitalization (LAPS2), service line (medicine vs. surgical), functional status (non-ambulatory, ambulatory with assistance, or ambulatory) and fall risk (Schmid score of 3+) within 24 h of discharge; disposition (home vs. home health), and hospital site

### Secondary subgroup analyses

For high risk (LACE 11+) medicine patients, both POSH (HR: 0.77, 95% CI: 0.71, 0.85, *P* < .001) and TPOSH (HR: 0.91, 95% CI: 0.83, 0.99, *P* = .03) provider visits were associated with 23 and 9% lower risk of 30-day readmission, respectively, compared to no visit in the weighted models. For medium to low risk patients (LACE< 11), neither POSH (HR: 0.84, 95% CI: 0.67, 1.05, *P* = .12) nor TPOSH (HR: 1.14, 95%CI: 0.94, 1.39, *P* = 0.19) provider visits were associated with lower readmission risk compared to no visit.

For all surgical patients regardless of LACE score or age, there were no significant associations for any visit type with risk of 30-day readmission in the weighted models.

## Discussion

Using a more contemporary and diverse cohort of patients discharged from hospital to home or home health, we found that completion of either a clinic or telephone provider follow-up visit within 7 days of discharge was associated with lower 30-day readmission compared to no visit *only* for high risk medicine patients. The risk reduction for POSH provider visits was comparable to our previous report [3] while the TPOSH effects though statistically significant, were marginal at best. A surprising finding was that none of the visit types were associated with lower readmission for lower risk medicine and all surgical patients.

Although the TPOSH was introduced to address a gap for patients who have mobility and transportation challenges and who would otherwise not have any follow-up provider visit, the limited effects of TPOSH compared to POSH were not surprising for several reasons. Clinic providers may have had insufficient practice with telephonic transitional care management prior to the pandemic as demonstrated in the small volume of TPOSH visits and may have been more cautious in directing patients to seek hospital-based care for worsening symptoms. Other ancillary team members were not able to augment the provider’s care as they would during a clinic visit. Neither physical exams nor visual assessments were performed during these encounters and thus providers were limited to patient and/or family self-report. With as many as half of elderly hospitalized patients having cognitive impairment [20], and possibly, without the assistance of a capable family member, gaps in communication and early detection of decompensation may be problematic. Use of video and/or remote assessments or biometrics could potentially enhance the effectiveness of these telephone encounters, especially for more complex, higher-risk older patients but only if family can assist with these technologies [21, 22].

The COVID-19 pandemic has accelerated adoption of digital technologies [7, 23] across all aspects of health care, especially with physically-distanced care and the earlier discharge of COVID-19 patients to increase hospital capacity [24]. Learnings from use of digital home-monitoring technologies for transitional care with COVID-19 patients could serve as exemplars for optimizing transitional care approaches for other medical conditions in the future. Since the start of the pandemic through December 2020, this health system conducted 87% of the transitional care management visits via telephone, 3% by video, and 10% in person, a complete flip compared to the pre-pandemic era. While there are no other published reports specifically on virtual care transition management practices, Eberly et al. [25] reported a more balanced distribution of video (45%) and telephone (55%) primary and specialty care visits from a large academic health system from March to May 2020. We expect that video visits will increase over time with greater provider comfort and increased patient access to technology [26] and also due to telehealth reimbursement for Medicare Advantage plans which took effect in January 1, 2020 but the long-term future of telehealth reimbursement in fee-for-service Medicare remains to be seen after the end of the public health emergency [23].

The finding that completion of any follow-up visit whether with a provider or ancillary care staff within 7 days of hospital discharge was not associated with lower readmission risk compared to no visit for surgical patients merit more detailed analyses by types of surgical procedures (high vs. low risk, planned vs. unplanned) to determine the optimal timing and appropriate type of provider follow-up post hospitalization in order to maximize value for health systems and payers [27]. In contrast to medicine patients, nearly half of the surgical patients did not have an outpatient follow-up visit within a week of discharge. It is possible that because the vast majority (87%) of these patients were considered as lower risk for readmission (LACE scores < 11), follow-up visits were rightly not prioritized by the discharging providers as our analyses showed that these visits were not associated with any added benefit in terms of risk reduction.

Strengths of this study included use of a large, diverse contemporary cohort that was exposed to many changes in the care transition management practices in recent years, replication of findings from our previous, limited analyses of an older study cohort, ability to balance relevant baseline covariates across comparison groups using matching weights, and inclusion of both inpatient and observation stay readmissions as a primary outcome in anticipation of changes to how readmissions are counted in the Center for Medicare and Medicaid Services Hospital Readmission Reduction Program as of January 1, 2021.

### Limitations

Since this analysis was limited to patients discharged from Kaiser Permanente hospitals to home, the findings may not generalize to patients discharged to other higher-level settings or in non-integrated care systems where electronic records are not easily shared across hospital and ambulatory care settings. The TPOSH volume was relatively small for several of the subgroups, especially for younger patients and those on the surgical service, to obtain a reliable estimate of benefit or harm. Since the care transition quality improvement efforts in this health system did not distinguish across disease states nor cause of the index admission except for medicine vs. surgical, we did not explore additional subgroup analyses in this paper though this should be the focus of future study. The matching weights were balanced on measured covariates but the cohorts may differ in other ways; omission of unmeasured confounders such as exposure to other care transition interventions and treatment adherence, as well selection bias, are other notable limitations of this observational study design. Nonetheless, prior studies of heart failure and COPD for instance, have also reported an association between completion of follow-up provider visits within 7 days post-discharge and lower rates of 30-day readmission [28, 29]. We did not account for the multiple comparisons in the subgroup analyses and thus some of the statistically significant findings may be spurious. However, we were cautious with our interpretation of these results. Finally, we were not able to assess the frequency of clinical escalations associated with the TPOSH visits.

## Conclusions

We found that completion of either a clinic or telephone provider follow-up visit within 7 days of hospital discharge was associated with lower 30-day inpatient or observation readmission compared to no visit *only* for high risk medicine patients. The effects of the telephone provider follow-up, though statistically significant, were marginal at best during the pre-pandemic era. It remains to be determined if greater use and comfort with virtual visits by providers and patients as a result of the pandemic might improve the effectiveness of these encounters. The dramatic transformation of care delivery to virtual care in response to the COVID-19 pandemic offers an unprecedented opportunity to further examine the right mix of in-person clinic or home-based and remote care (telephone, video, and e-visits) for post-hospital discharge follow-up across multiple relevant patient subgroups [30] to ensure equitable access to high quality care transition services [25, 31, 32].

## Data Availability

The datasets generated and/or analysed during the current study are not publicly available due to the identifiable nature of the data but may be available in anonymized form from the corresponding author under the following conditions: (1) agreement to collaborate with the study team on all publications, (2) provision of external funding to anonymize the data and for administrative and investigator time necessary for this collaboration, (3) demonstration that the external investigative team is qualified and has documented evidence of training for human subjects protections, and (4) agreement to abide by the terms outlined in data use agreements between institutions.
